# Drug-induced QT prolongation and torsade de pointes: a real-world pharmacovigilance study using the FDA Adverse Event Reporting System database

**DOI:** 10.3389/fphar.2023.1259611

**Published:** 2023-12-21

**Authors:** Dongxuan Li, Shuang Chai, Hongli Wang, Jie Dong, Chunmeng Qin, Dan Du, Yalan Wang, Qian Du, Songqing Liu

**Affiliations:** ^1^ Department of Pharmacy, The Third Affiliated Hospital of Chongqing Medical University, Chongqing, China; ^2^ Department of Pharmacy, The Affiliated Yongchuan Hospital of Chongqing Medical University, Chongqing, China; ^3^ College of Pharmacy, Chongqing Medical University, Chongqing, China

**Keywords:** QT prolongation, torsade de pointes, FDA adverse event reporting system, disproportionality analysis, pharmacovigilance, adverse reaction

## Abstract

**Introduction:** Drug-induced QT prolongation and (or) Torsade de Pointes (TdP) is a well-known serious adverse reaction (ADR) for some drugs, but the widely recognized comprehensive landscape of culprit-drug of QT prolongation and TdP is currently lacking.

**Aim:** To identify the top drugs reported in association with QT prolongation and TdP and provide information for clinical practice.

**Method:** We reviewed the reports related to QT prolongation and TdP in the FDA Adverse Event Reporting System (FAERS) database from January 1, 2004 to December 31, 2022, and summarized a potential causative drug list accordingly. Based on this drug list, the most frequently reported causative drugs and drug classes of QT prolongation and TdP were counted, and the disproportionality analysis for all the drugs was conducted to in detect ADR signal. Furthermore, according to the positive–negative distribution of ADR signal, we integrated the risk characteristic of QT prolongation and TdP in different drugs and drug class.

**Results:** A total of 42,713 reports in FAERS database were considered to be associated with QT prolongation and TdP from 2004 to 2022, in which 1,088 drugs were reported as potential culprit-drugs, and the largest number of drugs belonged to antineoplastics. On the whole, furosemide was the most frequently reported drugs followed by acetylsalicylic acid, quetiapine, citalopram, metoprolol. In terms of drug classes, psycholeptics was the most frequently reported drug classes followed by psychoanaleptics, analgesics, beta blocking agents, drugs for acid related disorders. In disproportionality analysis, 612 drugs showed at least one positive ADR signals, while citalopram, ondansetron, escitalopram, loperamide, and promethazine were the drug with the maximum number of positive ADR signals. However, the positive-negative distribution of ADR signals between different drug classes showed great differences, representing the overall risk difference of different drug classes.

**Conclusion:** Our study provided a real-world overview of QT prolongation and TdP to drugs, and the presentation of the potential culprit-drug list, the proportion of reports, the detection results of ADR signals, and the distribution characteristics of ADR signals may help understand the safety profile of drugs and optimize clinical practice.

## 1 Introduction

Drug-induced QT prolongation leading to torsade de pointes (TdP) is a type of cardiotoxic adverse reaction (ADR) mainly caused by the interference of drugs in the cardiac potassium current, which can be characterized by a “twisting of the points” around the isoelectric line and exaggerated prolongation of the QT interval on the electrocardiogram (Lazzara, 1997; Roden, 2016). In the general population, it is reported that the annual incidence of drug-triggered QT prolongation and TdP is estimated to be 2.5 per million for men and 4.0 per million for women (Sarganas et al., 2014). Although the incidence of such cardiotoxic ADR is very low, it can be life-threatening, and its mortality can reach an astonishing 10%–20% (Shah, 2013).

The “International Conference on Harmonization of Technical Requirements for Registration of Pharmaceuticals for Human Use” (ICH) issued the E14 clinical guidance in May 2005 to standardize the risk assessment and identification of QT prolongation of drugs before marketing, and it has currently become a standard component in new drug development programs (Darpo, 2010). However, although the safety of drugs has been strictly evaluated in clinical trials, these pre-marketing studies are usually limited to size and duration and exclude high-risk populations, so it is difficult to fully represent real-world populations and roundly detect rare but potentially life-threatening ADRs (Singh and Loke, 2012; Trifirò and Crisafulli, 2022). To facilitate a better understanding of the QT prolongation and TdP risk of drugs, the Arizona Center for Education and Research on Therapeutics (AZCERT) has summarized a drug list known as QTdrugs, which includes over 220 drugs and divides them into four risk categories based on its association with QT prolongation and TdP (Woosley et al., 2018). Undoubtedly, such a list highlights the drugs that need to be focused on and helps guide clinical management in patients who are at risk, exposed to QT-prolonging medication, or have QT prolongation (Page et al., 2016; Woosley et al., 2018; Khatib et al., 2021). However, the QTdrugs list only issued the risk information of fewer than 300 drugs, and some drugs with the potential risk of QT prolongation and TdP may not be identified and emphasized. Therefore, it is necessary to further comprehensively investigate and summarize the possible high-risk drugs related to QT interval prolongation and TdP.

Pharmacovigilance is the science and activities relating to the detection, assessment, understanding, and prevention of adverse effects or any other possible drug-related problems, and currently, using real-world data in pharmacovigilance databases to explore and summarize drug risk characteristics has become an important measure to evaluate drug safety (Beninger, 2018; Lucas et al., 2022). To some extent, it can break the inherent limitations of size, duration, and population selection in preclinical research and provide a real-time overview of main toxicities in a cost-effective manner, thereby providing information for clinical practice (Li et al., 2023a). In this respect, the FDA Adverse Event Reporting System (FAERS) database, a freely accessible pharmacovigilance database with massive real-world data and wide geographic coverage, provides an unprecedented opportunity to comprehensively investigate and summarize the risk of QT prolongation and TdP triggered by drugs.

In this study, we reviewed all the reports in the FAERS database that are associated with the occurrence of QT prolongation and TdP and conducted ADR signal detection for all the drugs that were reported as culprit-drugs of QT prolongation and TdP using disproportionality analysis, aiming at providing a comprehensive overview of drugs that potentially induced QT prolongation and TdP from the pharmacovigilance perspective and informing clinical practice.

## 2 Methods

### 2.1 Data source

This pharmacovigilance study was carried out based on the FAERS database, which contained post-marketing adverse event (AE) reports on drugs and therapeutic biologic products submitted by healthcare professionals, consumers, and manufacturers. At present, the FAERS database has published more than 16 million AE reports received by the FDA since 2004 on the openFDA website (https://open.fda.gov/apis/drug/), and the data are updated quarterly. The data recorded in the AE report mainly consist of seven parts: patient demographic information, drug information, adverse event information, patient outcome information, report source information, drug therapy date information, and drug indication (Cirmi et al., 2020). Those data are highly structured, so they can be retrieved and downloaded by constructing a reasonable retrieval formula through the application program interface (API) (Kass-Hout et al., 2016).

### 2.2 Determination of reports of interest

In FAERS, AE-related information is standardized to preferred terms (PTs) using the Medical Dictionary for Regulatory Activities (MedDRA) (Brown, 2003). Standardized MedDRA Queries (SMQs) are a group containing multiple PTs, which represent signs, symptoms, diagnoses, syndromes, physical findings, and laboratory and other physiological test data likely to be relevant to the medical condition of interest (Mozzicato, 2007). There are two types of applications for most SMQs, namely, narrow-scope search and broad-scope search. The narrow-scope search consists of PTs that have no reasonable doubt about the medical condition of interest, while the broad-scope search contains PTs of the narrow search and the PTs that could be related to the medical condition of interest but have some uncertainty (Cirmi et al., 2020). In order to ensure the accuracy of target event recognition, in this study, only PTs contained in the narrow-scope search of “torsade de pointes/QT prolongation (SMQs)” in MedDRA 23.0 were used to identify target AE cases (Table 1).

**TABLE 1:** Preferred terms (PTs) contained in the narrow-scope search of “torsade de pointes/QT prolongation (SMQs).”

Preferred term	MedDRA code
Electrocardiogram QT prolonged	10014387
Ventricular tachycardia	10047302
Torsade de pointes	10044066
Long QT syndrome	10024803
Electrocardiogram QT interval abnormal	10063748
Long QT syndrome congenital	10057926
Torsade de pointes/QT prolongation (SMQs)^a^	20000001

^a^ This is an SMQ term which includes six preferred terms in the narrow-scope search. MedDRA, Medical Dictionary for Drug Regulatory Activities; SMQs, Standardized MedDRA Queries.

### 2.3 Adverse reaction signal detection method

The reporting odds ratio (ROR) is a classic disproportionality analysis method widely used in detecting ADR signals (Sakaeda et al., 2013). The principle of the ROR method is to compare the drug exposure of cases of an AE of interest with that of cases with other reported AEs, thus reflecting the degree of correlation between the target drug and target AE (Faillie, 2019). According to the two-by-two contingency table (Table 2), the ROR value and its corresponding 95% confidence intervals (CIs) can be calculated using the following equations:

$$\text{ROR}=\frac{a/c}{b/d}=\frac{ad}{bc},$$ (1)

$$95\%\mathrm{CI}=e^{\ln\left(ROR\right)\;\pm 1.96\sqrt{\left(\frac{1}{a}+\frac{1}{b}+\frac{1}{c}+\frac{1}{d}\right)}}.$$ (2)

**TABLE 2:** Two-by-two contingency table for disproportionality analysis.

	Drug of interest	Other drugs	Total
AE of interest	a	b	a + *b*
Other AEs	c	d	c + d
Total	a + c	b + d	a + *b* + c + d

AE, adverse event.

Referring to the number of cases and the value of the lower limit of 95% CI, the ADR signal detection results can be further classified into negative and positive signals. A signal is considered positive when there are at least three cases (a ≥3 in Table 2) and the lower limit of 95% CI > 1, while a signal is considered negative when the number of cases or the lower limit of 95% CI cannot meet the aforementioned criteria (Li et al., 2023a).

### 2.4 Data processing and analysis

Referring to the API retrieval construction instructions of openFDA (https://open.fda.gov/apis/drug/event/how-to-use-the-endpoint/), the retrieval and downloading of AE reports can be realized by using the API. The returned data are in the form of a structured dataset stored in JSON format, which is convenient for further data processing and analysis. The detailed data processing and analysis steps of this study are as follows:

First, the PTs in Table 1 were used to call the API and download all the AE reports associated with QT prolongation and TdP from 1 January 2004 to 31 December 2022 from the FAERS database. If one of the PTs in Table 1 is recorded in the “patient.reaction.reactionmeddrapt” field of the AE report, we consider it a target AE report that is related to QT prolongation and TdP.

Second, the R packages “jsonlite” and “dplyr” were used to read and sort out information recorded in the downloaded dataset, including safety report ID number, patient demographic information, report years, report sources, drug use, and AE outcomes.

Third, pharmacists reviewed the generic names of the primary suspect drugs (“patient.drug.drugcharacterization” field = 1) recorded in the “patient.drug.openfda.generic_name” field in AE reports and coded the primary suspect drug based on the Anatomical Therapeutic Chemical (ATC) classification system, obtaining a final drug list with ambiguous drug names removed and synonymous drug names integrated.

Fourth, categorical statistics were conducted to summarize the top 10 drugs and ATC drug classes (second ATC level) with the highest reporting proportions in the PT and SMQ levels.

Fifth, based on the above drug list, ADR signal detection was performed on each drug at the PT and SMQ levels, yielding seven signal detection results (one for the SMQ level and six for the PT level).

Finally, based on the signal detection results at the PT and SMQs levels, the number of positive signals of each drug and the positive–negative distribution characteristics of ADR signals were summarized and integrated.

In this study, all the data processing and analyses were conducted using R version 4.1.0 (R Foundation for Statistical Computing, Vienna, Austria).

## 3 Results

### 3.1 Basic information of AE reports

**FIGURE 1:**
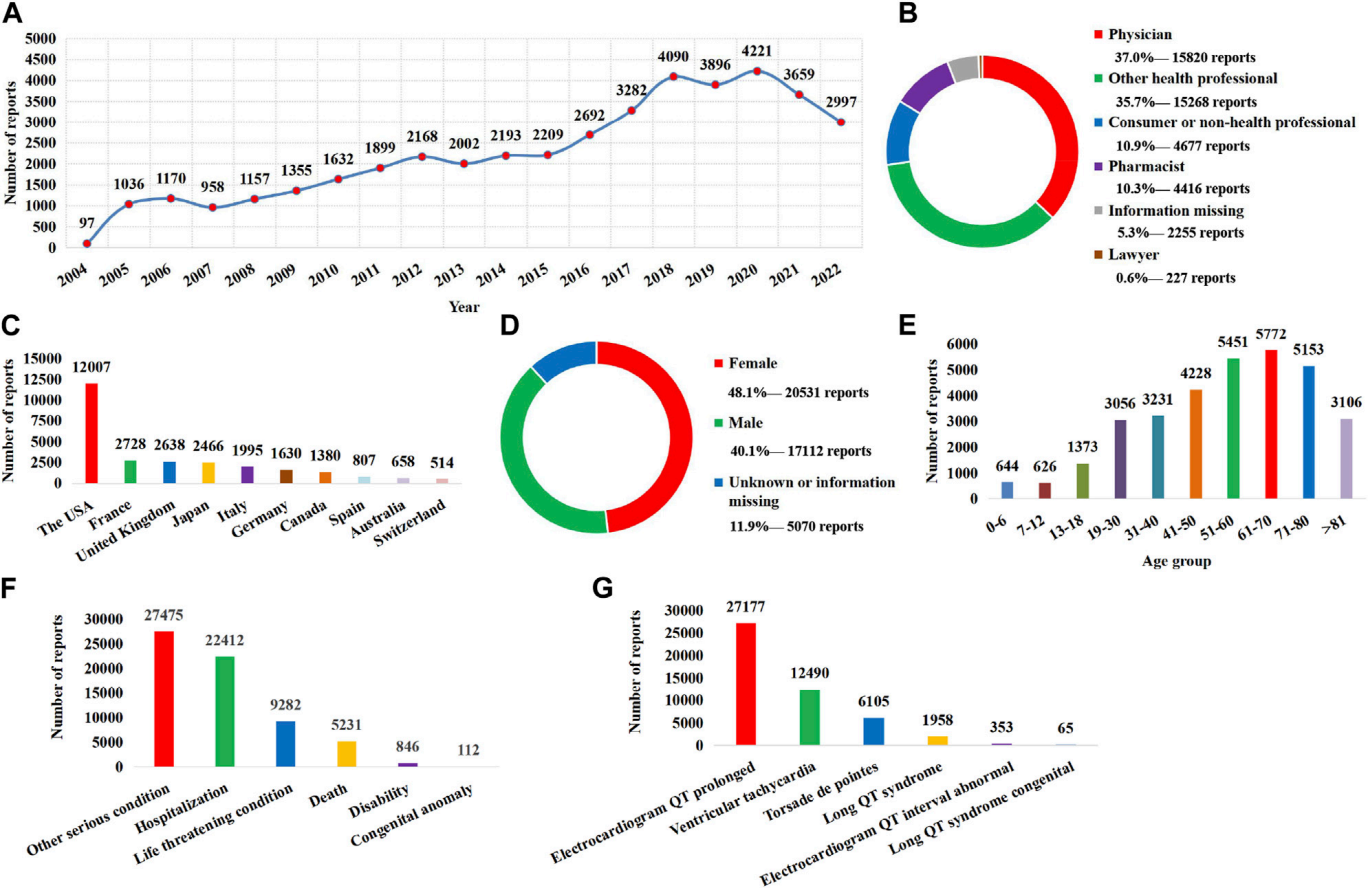
Basic information and clinical characteristics of the report associated with QT prolongation and torsade de pointes. **(A)** Distribution of the reporting years. **(B)** Distribution of report submitters. **(C)** Top 10 countries with the largest number of reports. **(D)** Sex distribution of patients. **(E)** Age distribution of patients. **(F)** Distribution of patient outcome. **(G)** Distribution of adverse event of preferred terms (PTs).

From 1 January 2004 to 31 December 2022, a total of 16,010,899 AE reports were included in the FAERS database, among which 42,713 were identified as target AE reports using the narrow-scope search of “torsade de pointes/QT prolongation (SMQs).” The annual distribution of AE reports related to QT prolongation and TdP is shown in Figure 1A, in which 2020 had the highest number of reports received. In terms of source and type of the report, health professionals (37.0% for physicians, 35.7% for other health professionals, and 10.3% for pharmacists) were the main submitters (Figure 1B), and the AE reports were mainly from the United States (Figure 1C). In terms of patients, the rate of women was higher than that of men (Figure 1D), and 61–70 years was the age group with the largest number of cases (Figure 1E). With regard to patient outcomes, hospitalizations accounted for 52.5% of cases, while death accounted for 12.2% of cases (Figure 1F). In the narrow-scope search of “torsade de pointes/QT prolongation (SMQs),” electrocardiogram QT prolonged is the PT involving the largest number of AE reports (Figure 1G).

### 3.2 Determining the drug list associated with QT prolongation and TdP

**FIGURE 2:**
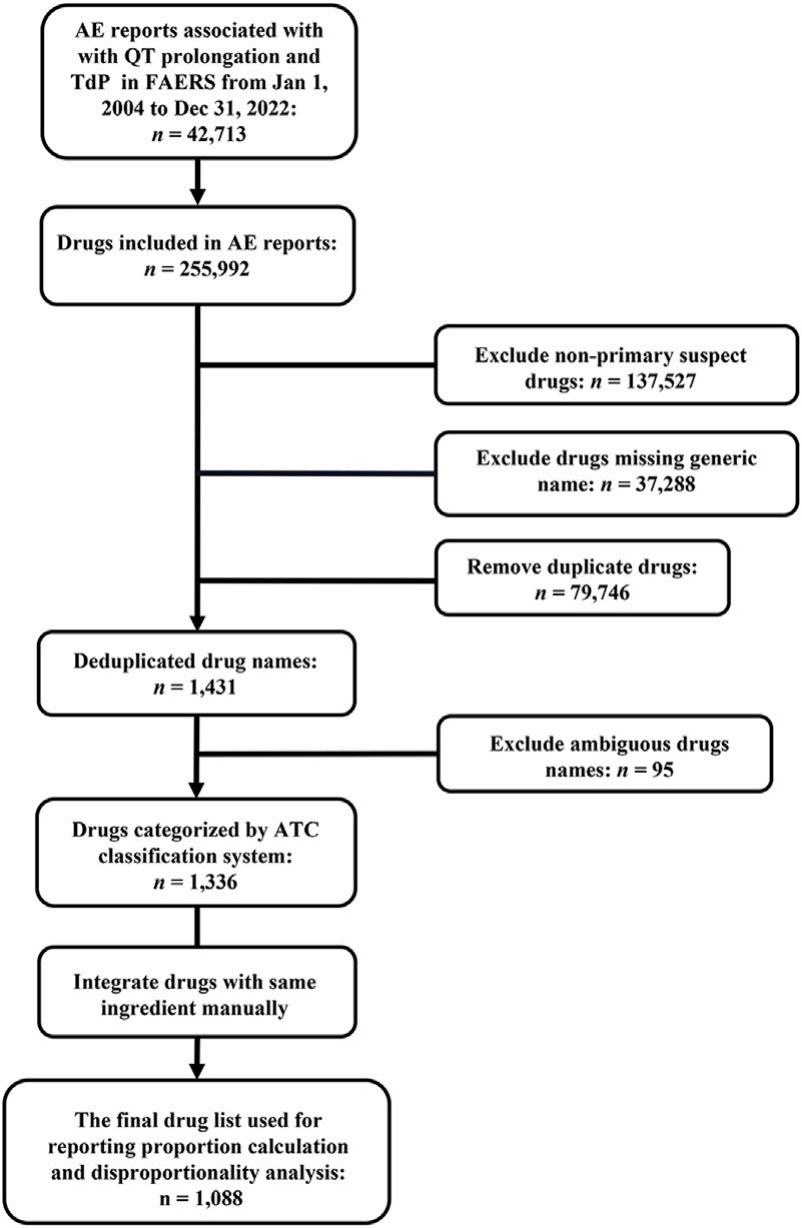
Flowchart of target report identification and potential culprit-drug summarization. AE, adverse event; ATC, Anatomical Therapeutic Chemical; FAERS, FDA Adverse Event Reporting System; TdP, torsade de pointes.

Due to an AE report usually listing multiple drugs that may be responsible for an AE of interest, there were a total of 255,992 drugs exposed to patients in 42,713 target AE reports during target AE occurrence. To obtain a final drug list to summarize the distribution of the culprit-drug and conduct ADR signal detection, non-primary suspect drugs (*n* = 137,527), drugs missing generic names (*n* = 37,288), and duplicate drugs (*n* = 79,746) were excluded. After that, the drug list was checked by a professional pharmacist to exclude drugs with ambiguous names (*n* = 95) and integrate drugs with the same active ingredient (e.g., acetaminophen and paracetamol). Finally, we obtained a drug list containing 1,088 drugs (Figure 2), each of which was considered to be responsible for the target AE occurrence in at least one report. Further associating 1,088 drugs with specific PT, the electrocardiogram QT prolonged (PT) group contained the largest number of drugs, while the long QT syndrome congenital (PT) group included the least number of drugs (Table 3).

**TABLE 3:** Number of drugs associated with the SMQ group and PT subgroup.

Group	No. (%) of drugs
Electrocardiogram QT prolonged	983 (90.3)
Ventricular tachycardia	966 (88.8)
Torsade de pointes	675 (62.0)
Long QT syndrome	524 (48.2)
Electrocardiogram QT interval abnormal	276 (25.4)
Long QT syndrome congenital	52 (4.8)
Torsade de pointes/QT prolongation (SMQs)^a^	1,088 (100.0)

^a^ This is an SMQ term, which includes six preferred terms in the narrow-scope search. SMQs, Standardized MedDRA Queries.

### 3.3 Proportional distribution of drugs in AE reports

**FIGURE 3:**
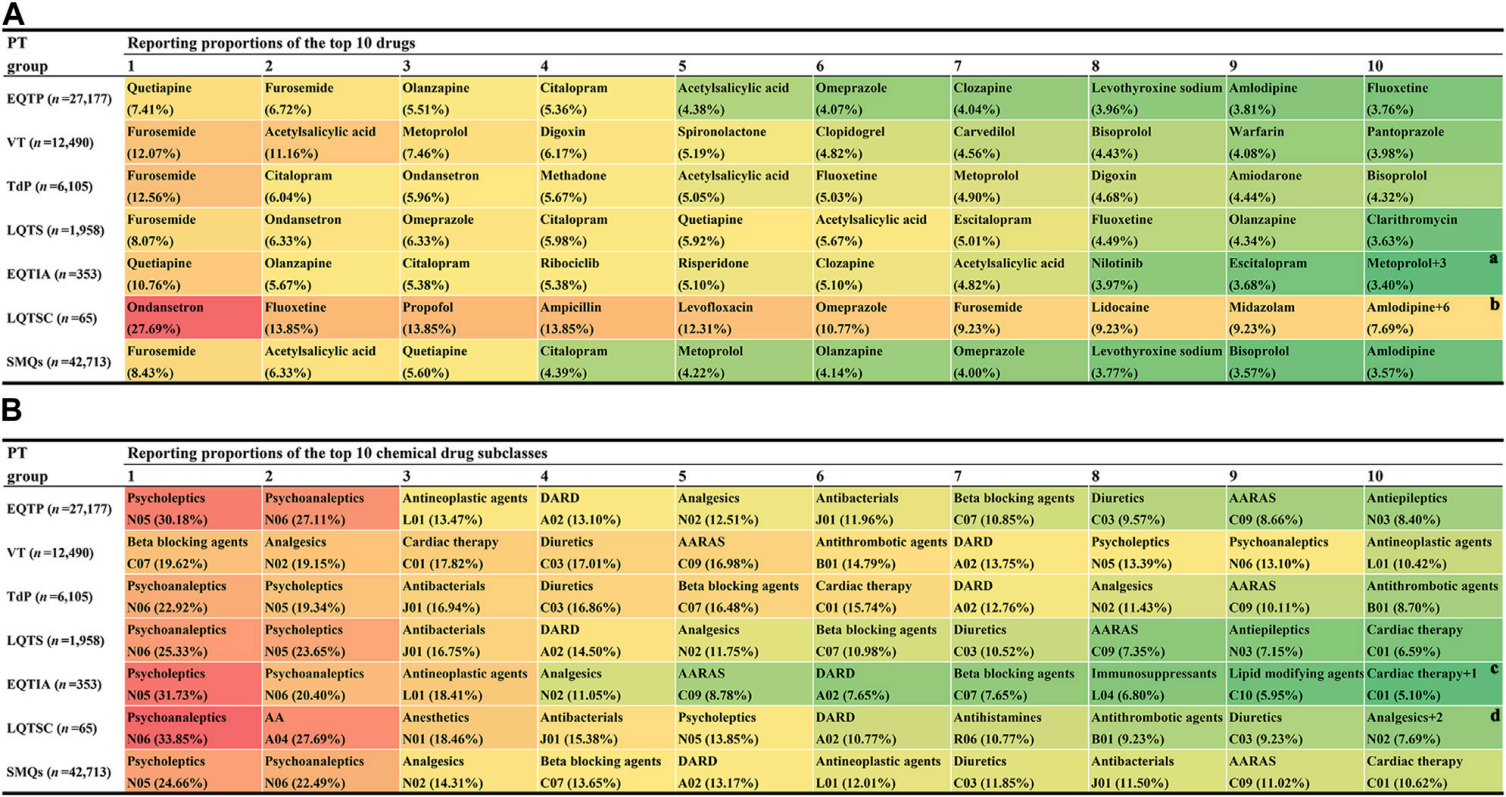
Proportional distribution of drugs associated with QT prolongation and torsade de pointes in adverse event reports. **(A)** Top 10 highest reporting proportion agents at the Standardized MedDRA Queries (SMQs) and preferred term (PT) levels. **(B)** Top 10 highest reporting proportion drug classes at the SMQ and PT levels. AA, antiemetics and antinauseants; AARAS, agents acting on the renin–angiotensin system; DARD, drugs for acid-related disorders; EQTIA, electrocardiogram QT interval abnormal; EQTP, electrocardiogram QT prolonged; LQTS, long QT syndrome; LQTSC, long QT syndrome congenital; TdP, torsade de pointes; VT, ventricular tachycardia. ^a^ There are three other drugs (lorazepam, lithium, and fingolimod) with the same reporting proportion as that of metoprolol. ^b^ There are six other drugs (bupropion, promethazine, warfarin, fentanyl, alprazolam, and methylphenidate) with the same reporting proportion as that of amlodipine. ^c^ There is one other drug class, antiepileptics (N03) with the same reporting proportion as that of cardiac therapy. ^d^ There are two other drug classes, muscle relaxants (M03) and calcium channel blockers (C09) with the same reporting proportion as that of analgesics (N02).

Based on the counts of AE reports, the reporting distribution of 1,088 drugs was summarized. The top 10 most frequently reported drugs at the SMQ and PT levels are shown in Figure 3A. On the whole (at the SMQ level), furosemide (8.43%) was the most frequently reported drug, followed by acetylsalicylic acid (6.33%), quetiapine (5.60%), citalopram (4.39%), metoprolol (4.22%), olanzapine (4.14%), omeprazole (4.00%), levothyroxine sodium (3.77%), bisoprolol (3.57%), and amlodipine (3.57%). Using the ATC classification system, the 1,088 drugs were classified as the second ATC level. Similarly, according to the counts of AE reports, the top 10 most frequently reported drug classes at the SMQ and PT levels are shown in Figure 3B. On the whole (at the SMQ level), psycholeptics (24.66%) were the most frequently reported drug class, followed by psychoanaleptics (22.49%), analgesics (14.31%), beta-blocking agents (13.65%), drugs for acid-related disorders (13.17%), antineoplastic agents (12.01%), diuretics (11.85%), antibacterials (11.50%), agents acting on the renin–angiotensin system (11.02%), and cardiac therapy (10.62%).

### 3.4 ADR signal detection results

To evaluate the potential risk of QT prolongation and TdP for 1,088 drugs, each drug in the list was combined with PT and SMQs in Table 1, respectively, to conduct disproportionality analysis, namely, yielding seven ADR signals for each drug (one for the SMQ level and six for the PT level). Details of the ADR signal detection results of 1,088 drugs are shown in Supplementary Table S1.

**FIGURE 4:**
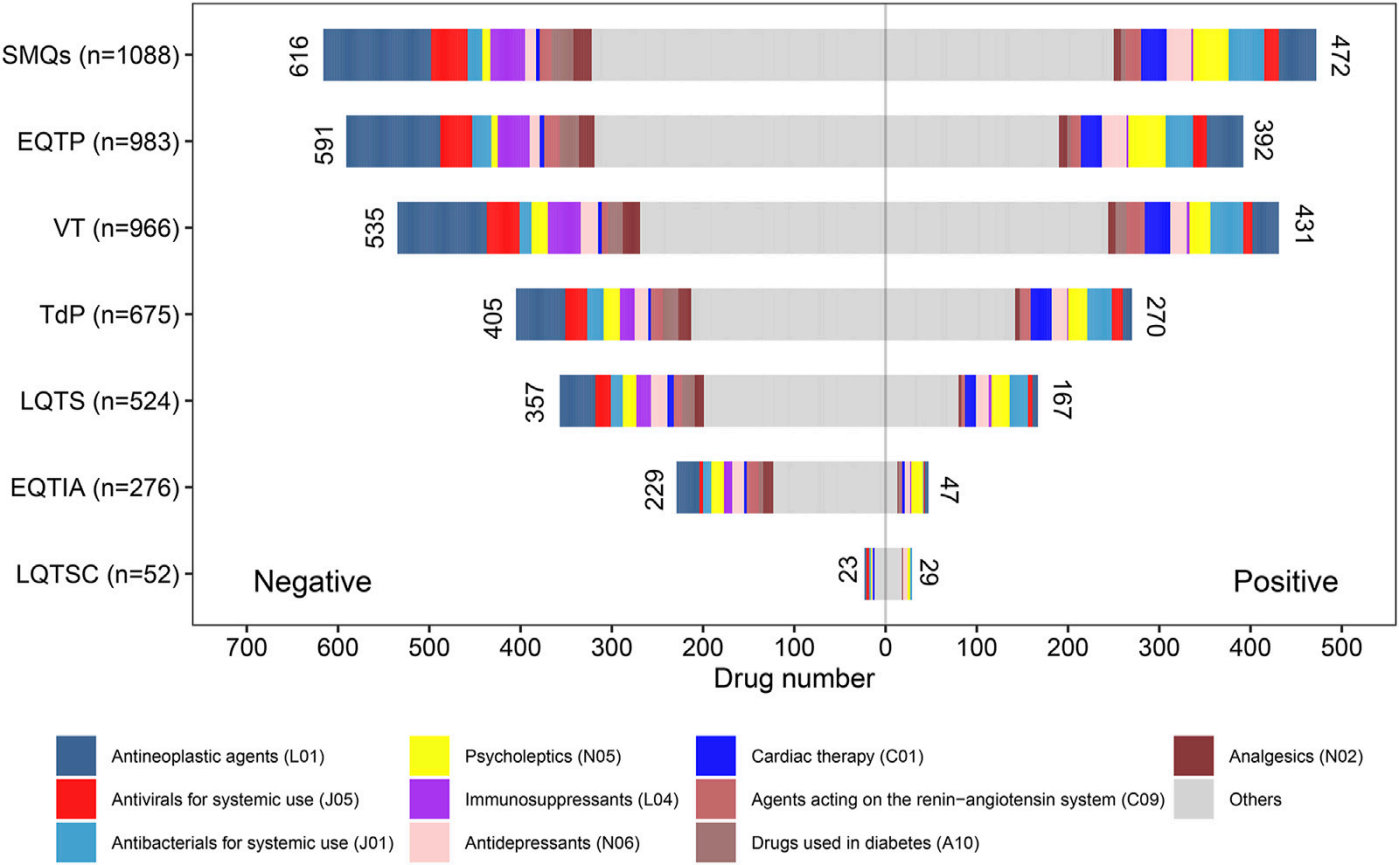
Drug class distribution of potential culprit-drugs and the positive–negative distribution of adverse reaction signals at the Standardized MedDRA Queries (SMQs) and preferred term (PT) levels. EQTIA, electrocardiogram QT interval abnormal; EQTP, electrocardiogram QT prolonged; LQTS, long QT syndrome; LQTSC, long QT syndrome congenital; TdP, torsade de pointes; VT, ventricular tachycardia.

For the SMQ-level group and each PT-level group, the positive–negative distribution of ADR signals of drugs and the corresponding drug category distribution are summarized in Figure 4. On the whole, there were more negative-signal drugs than positive-signal drugs in most groups. In terms of the drug class (ATC second level), antineoplastic agents (L01), antivirals for systemic use (J05), antibacterials for systemic use (J01), psycholeptics (N05), immunosuppressants (L04), antidepressants (N06), cardiac therapy (C01), agents acting on the renin–angiotensin system (C09), drugs used in diabetes (A10), and analgesics (N02) were the top ten suspicious causative drug classes involved in most groups. However, it is noteworthy that there was a big difference in ADR signal distribution between different drug classes.

To further summarize the risk characteristics of 1,088 drugs, we integrated the total number of positive signals of each drug. For each drug that underwent disproportionality analysis, the sum of the number of positive signals may be between 0 and 7, and the positive-signal number distribution of 1,088 drugs is shown in Table 4. Among 1,088 drugs, 476 (43.8%) drugs did not show any positive ADR signal. Among the drugs that had at least one positive ADR signal, five drugs (citalopram, ondansetron, escitalopram, loperamide, and promethazine) contained the maximum number of positive ADR signals, i.e., each drug contained seven positive ADR signals. The sum of the number of positive signals of each drug is listed in Supplementary Table S1.

**TABLE 4:** Distribution of the number of positive ADR signals.

Number of positive ADR signals	No. (%) of drugs
7	5 (0.5%)
6	38 (3.5%)
5	88 (8.1%)
4	98 (9.0%)
3	117 (10.8%)
2	137 (12.6%)
1	129 (11.9%)
0	476 (43.8%)
Total	1,088 (100.0%)

ADR, adverse reaction.

## 4 Discussion

Drug-induced QT prolongation and TdP is an interdisciplinary drug safety issue that has received much attention, which can result in sudden cardiac death. This study comprehensively evaluated the AE reports of drug-induced QT prolongation and TdP in the real world based on the FAERS database. We described the basic characteristics of the AE report of QT prolongation and TdP and summarized a list containing 1,088 drugs that were reported as the potential culprit-drugs of QT prolongation and TdP. Meanwhile, based on this drug list, we made statistics on the reporting proportion of different drugs and drug classes and conducted ADR signal detection and signal distribution integration for each drug.

In this study, a drug list containing all the primary suspected culprit-drugs of QT prolongation and TdP in FAERS was provided. To the best of our knowledge, this list, which contained 1,088 potential causative drugs of QT prolongation and TdP, is the most comprehensive list summarized using a pharmacovigilance database so far. Although previous studies have tried to use a pharmacovigilance database to explore and summarize high-risk drugs associated with QT prolongation and TdP (Poluzzi et al., 2010; Teng et al., 2019; Cirmi et al., 2020; Ali et al., 2021; He et al., 2021; Wu et al., 2022; Yu and Liao, 2022; Chen et al., 2023), the list provided by these studies is not comprehensive enough. The most related studies only pay attention to a certain drug class and, on this basis, evaluate and compare the risks of limited drugs, such as H1-antihistamines (Ali et al., 2021), antifungal triazoles (Yu and Liao, 2022), antibacterial drugs (Teng et al., 2019), antipsychotics (He et al., 2021), tyrosine kinase inhibitors (Cirmi et al., 2020), and antidepressants (Chen et al., 2023). Although those studies highlighted the drugs worthy of attention in the same category, it is difficult to rationally integrate them into a list because of the difference in data time included, inclusion and exclusion criteria of AE reports, and ADR signal detection methods. In order to overcome those limitations, Wu et al. (2022) investigated and evaluated all risky drugs associated with TdP according to the FAERS database with a unified standard. However, it only selected one of the PTs (torsade de pointes, MedDRA code: 10044066) in “torsade de pointes/QT prolongation (SMQs)” to identify target AE reports and only showed the top 50 most frequently reported drugs and the top 50 risky drugs with the highest ADR signal strength. As shown in Table 1, QT prolongation and TdP are medical conditions consisting of a series of closely related PTs, which means that “torsade de pointes (PT)” can only be used to identify part of AE reports associated with QT prolongation and TdP and summarize part of potential causative drugs related to QT prolongation and TdP (Figure 1G; Table 3). Therefore, the limitation of target AE report identification and drug display cannot fully support it to provide a precise and comprehensive drug list for QT prolongation and TdP. In our study, the narrow search of “torsade de pointes/QT prolongation (SMQs)” was used to identify target AE reports, which greatly ensured the rationality and completeness of our drug list.

In addition to offering a complete drug list, this study also provided a multi-dimensional evaluation perspective. First, we showed the top 10 most frequently reported drugs and drug classes at the SMQ and PT levels, respectively, to locate the drugs and drug classes worthy of attention. For example, furosemide was the most frequently reported drug at the SMQ level in our study. Previous studies have shown that exposure to furosemide is a risk factor for QT prolongation and TdP, and the potential mechanism underlying it may be related to the electrolyte disorder caused by furosemide (Drew et al., 2010). Similarly, psycholeptics (N05) were the most frequently reported drugs at the SMQ level in our study, and previous studies have also proven that psycholeptics are closely related to QT prolongation and TdP (Beach et al., 2013). Therefore, according to this report’s proportion result, we can use it to quickly understand the drugs and drug classes that commonly result in QT prolongation and TdP in the real world. However, it is worth noting that a high reporting ratio does not always represent a high risk because, for different drugs, the frequency of drug use will vary greatly, which will directly affect the proportion of ADR reports.

Based on the reasons mentioned above, we introduced the disproportionality analysis method as a uniform standard to evaluate the risk of QT prolongation and TdP of drugs. Although previous studies have used similar ADR signal mining methods to explore the QT prolongation and TdP risk of drugs, the scope of those research studies mainly focused on specific drug classes and specific PTs (Poluzzi et al., 2010; Teng et al., 2019; Cirmi et al., 2020; Ali et al., 2021; He et al., 2021; Wu et al., 2022; Yu and Liao, 2022; Chen et al., 2023). Therefore, it is difficult to use the results of those studies to reasonably compare the QT prolongation and TdP risks of drugs in different drug categories. In our study, based on a comprehensive drug list containing 1,088 drugs, the ADR signals at the SMQ and PT levels were thoroughly detected (Supplementary Table S1), which eliminated the obstacles of cross-drug class and cross-PT risk comparison. In addition, in order to present the risk characteristics of a drug as a whole (Li et al., 2023a; Li et al., 2023b), the sum of the number of positive signals for each drug was calculated. Among the 1,088 drugs, 612 drugs have at least one positive ADR signal, which suggests that we need to pay attention to the QT prolongation and TdP risk of these drugs, especially those with multiple positive ADR signals. For example, the drugs with seven positive ADR signals, citalopram (Beach et al., 2013; Fung et al., 2021), ondansetron (Hafermann et al., 2011; Lee et al., 2017), escitalopram (Fung et al., 2021), loperamide (Swank et al., 2017), and promethazine (Ali et al., 2021), have been reported as potential high-risk drugs for QT prolongation and TdP. In this connection, if we use this index reasonably, it can be used as a quick tool to understand the risk characteristics of a certain drug and provide its safety information.

Furthermore, based on the results of drug ADR signal detection, we also paid special attention to the positive–negative distribution of ADR signals across different drug categories at the SMQ and PT levels (Figure 4). On the whole (at SMQs level), antineoplastic agents (L01) are the drug class involving the largest number of drugs reported as potential culprit-drugs, and it is also the drug class that contains the largest number of drugs with positive ADR signals. Although previous studies have recognized the potential association between antineoplastic agents and cardiac rhythm disorders (Alexandre et al., 2018; Roden, 2019; Salem et al., 2021) and put forward corresponding risk evaluation and management measures (Sarapa and Britto, 2008; Coppola et al., 2018), the status of antineoplastic agents was not prominent among various risk drug classes. Our results suggest that antineoplastic agents have become the top drug class that trigger QT prolongation and TdP, so we should pay more attention to the heart safety of antineoplastic drugs, especially under the current background concerning the development and clinical application of antineoplastic drugs. Following antineoplastic agents (L01), antivirals for systemic use (J05), antibacterials for systemic use (J01), psycholeptics (N05), and immunosuppressants (L04) were the drug classes with the largest number of drugs that were reported to trigger QT prolongation and TdP. However, it is noteworthy that there was a big difference in ADR signal positive–negative distribution among the above-mentioned drug classes, in which most drugs in antibacterials for systemic use (J01) and psycholeptics (N05) showed a positive ADR signal, while most drugs in antivirals for systemic use (J05) and immunosuppressants (L04) showed a negative ADR signal. To some extent, such a difference in ADR signal positive–negative distribution can be explained by the risk difference of QT prolongation and TdP in different drug classes. In the previous literature, the risk of QT prolongation and TdP is well-recognized and prominent in antibacterials and psycholeptics (Straus et al., 2004; Sicouri and Antzelevitch, 2008; Ray et al., 2009; Abo-Salem et al., 2014), but such a risk is undefined in antivirals and immunosuppressants, which means that the overall QT prolongation and TdP risk of antibacterials and psycholeptics may be higher than that of antivirals and immunosuppressants. In this regard, our study provided a landscape to understand and compare the overall risk of different drug categories.

We acknowledge that our study also has some inherent limitations. First, the true incidence of QT prolongation and TdP with the use of drugs cannot be evaluated because the exact denominator of patients exposed to each drug is unknown. Second, due to the voluntary nature of reporting to FAERS, AE reports with variable degrees of exhaustivity may have an uncertain influence on the result. Third, multiple factors, such as the extent of use of the product, publicity, the nature of the reactions, underreporting, Weber effect, and notoriety bias (Hoffman et al., 2014; Alatawi and Hansen, 2017; Bihan et al., 2020; Neha et al., 2021), may influence the final number of AE reports for a particular drug and drug class, thereby causing a deviation on report proportion statistics and ADR signal detection. Forth, many factors, including sex, age, drug–drug interactions induced by concomitant drugs (Kim et al., 2020), dosage and duration of drugs used, and complications of the patients, may potentially affect the occurrence of QT prolongation and TdP. However, it is almost impossible to shield the potential interference of those confounding factors to the ADR detection results due to the inherent limitations of the pharmacovigilance database, so it is necessary to further investigate the potential influence of these factors on the occurrence of QT prolongation and TdP in a well-designed study. Finally, the results of ADR signal detection only reflect the statistical correlation between the target drug and the target AE, and all hypotheses generated require validation by translational mechanistic or prospective studies.

## 5 Conclusion

Based on the review of the publicly available FAERS data, our study summarized a comprehensive potential culprit-drug list of QT prolongation and TdP, obtained the statistics of the most frequently reported causative drugs and drug classes of QT prolongation and TdP, conducted ADR signal detection, and integrated the ADR signal detection results. To some extent, our study provided a preliminary whole picture of the potential culprit-drugs for QT prolongation and TdP in the real world, which can help regulators, health professionals, and others involved in drug management better understand the risk of QT prolongation and TdP for different drugs and optimize clinical practice. However, our study also has many limitations due to the nature of the pharmacovigilance database. It is particularly noteworthy that ADR signals only represent a statistical relationship between drugs and AE, and the real causal relationship between them needs further verification in a well-designed study. Therefore, in clinical practice, ADR signals can only be used as reference evidence and cannot replace the professional opinions of cardiologists and (or) clinical pharmacists.

## Data Availability

The original contributions presented in the study are included in the article/Supplementary Material; further inquiries can be directed to the corresponding authors.
